# Stromal–epithelial interactions influence prostate cancer cell invasion by altering the balance of metallopeptidase expression

**DOI:** 10.1038/sj.bjc.6601717

**Published:** 2004-03-30

**Authors:** L A Dawson, N J Maitland, A J Turner, B A Usmani

**Affiliations:** 1 Proteolysis Research Group, School of Biochemistry & Microbiology, University of Leeds, Leeds LS2 9JT, UK; 2 YCR Cancer Research Unit, Department of Biology, University of York YO10 5YW, UK

**Keywords:** prostate cancer, stromal–epithelial interactions, endothelin-1, endothelin-converting enzyme (ECE), neutral endopeptidase (NEP), invasion

## Abstract

Perturbations of stromal–epithelial interactions in the developing tumour can contribute to cancer invasion and metastasis. The structurally related metallopeptidases endothelin-converting enzyme (ECE) and neutral endopeptidase (NEP) contribute sequentially to the synthesis and inactivation of ET-1, a mitogenic peptide that has been shown to affect tumour behaviour. This study has investigated the interaction between metastatic tumour epithelial cells, which lack NEP, and stromal cells, which we have shown to express ECE-1 (stromal–epithelial interactions), using Matrigel invasion chambers. The epithelial cell lines utilised in this study include androgen-sensitive LNCaP, androgen-independent PC-3, Du145 and recently established PNT-1a, PNT2-C2 and P4E6 prostate cell lines. Specific inhibition of endogenous ECE-1 activity in stromal cells reduced PC-3 and Du145 invasion by 70 and 50%, respectively. Addition of recombinant NEP to inactivate endogenous mitogenic peptides resulted in 50 and 20% reductions in invasion in PC-3 and Du145 cells, respectively. Neutral endopeptidase effects were reversed in the presence of thiorphan, a specific NEP inhibitor. Supplementation of defined media with bradykinin and ET-1 significantly increased PC-3 invasion by 40 and 50%, respectively. Du145 cell invasion increased by approximately 100% on adding ET-1. These studies implicate the metallopeptidases NEP and ECE-1 as mediators of prostate cancer invasion via a stromal/epithelial interaction.

The majority of tumour studies have focused on events occurring within the malignant epithelial cells, which result in the disruption of molecular pathways regulating essential functions such as proliferation and invasion. More recently, studies have begun to document changes in the stromal environment surrounding the tumour (‘reactive stroma’) and its importance in promoting invasion, progression and metastasis (Tuxhorn *et al*, 2001). Human cancers have been found to generate a reactive stroma, which can promote tumorigenesis and influence epithelial cell invasion during *in vitro* modelling of prostate cancer invasion (Lang *et al*, 2000). Stromal cells derived from malignant prostate tissue can provide a greater stimulus for tumour cell invasion than their nonmalignant counterparts, but nonmalignant stromal cells can also influence the development and rate of human prostate tumorigenesis in mouse xenograft models (Tuxhorn *et al*, 2002).

Clinical and preclinical data have indicated a mitogenic role for small regulatory peptides such as endothelin-1 (ET-1), bradykinin, bombesin-like peptides and neurotensin in various stages of prostate cancer (Bologna *et al*, 1989; Papandreou *et al*, 1998). Endothelin-1, in particular, has been demonstrated in prostatic tissue *in vivo* and in human PC lines *in vitro* with plasma ET-1 concentrations significantly elevated in men with metastatic disease (Nelson *et al*, 1995). Endothelin-1 can also synergise the proliferative effects of other peptide growth factors in various prostate cancer cell lines (Battistini *et al*, 1993). Active ET-1 is generated from big-ET by endothelin-converting enzyme (ECE-1), which can also inactivate bradykinin (Hoang and Turner, 1997). Endothelin-converting enzyme-1 is a membrane-bound zinc-dependent metallopeptidase of the M13 family, which also includes the closely related peptidase, neutral endopeptidase (NEP, CALLA, CD10) (Turner *et al*, 2001). Endothelin-converting enzyme-1 has a broad tissue distribution and exists as four distinct isoforms termed ECE-1a, ECE-1b, ECE-1c (Schweizer *et al*, 1997) and ECE-1d (Valdenaire *et al*, 1999). These isoforms differ only in their N-terminal regions and are derived from a single gene through the use of alternative promoters. They have distinct subcellular locations: ECE-1a and ECE-1c are found at the cell surface, whereas ECE-1b and ECE-1d are reported to be located on intracellular membrane compartments (Muller *et al*, 2003).

Neutral endopeptidase inactivates a range of regulatory peptides, including ET-1 at the cell surface (Turner *et al*, 2001). Neutral endopeptidase is expressed on benign prostate epithelial cells but dramatically downregulated in metastatic human PC (Papandreou *et al*, 1998), and contributes to the transition of hormonally regulated androgen-dependent to independent PC (Papandreou *et al*, 1998) both via enzymatic activity and by nonenzymatic signalling interactions (Papandreou *et al*, 1998; Sumitomo *et al*, 2000).

We have recently reported an inverse correlation between NEP and ECE-1 expression in metastatic PC cell lines (Usmani *et al*, 2002). This study addresses the invasion-promoting properties of stromal cells on epithelial components of prostate cancer as a consequence of ECE and NEP expression. The purpose of this study was to investigate the relevance of stromal ECE-1 expression on the invasion of PC cells through Matrigel, by using stromal–epithelial co-culture techniques, and to examine whether invasion can be modulated using inhibitors of NEP and ECE-1.

## MATERIALS AND METHODS

### Materials

The ECE-1 monoclonal antibody AEC 32-236, which recognises all ECE-1 isoforms (described by Shimada *et al*, 1994), was generously donated by Dr K Tanzawa (Sankyo Research Laboratories, Tokyo). The NEP monoclonal (NCL-CD10-270) was obtained from Novocastra (Newcastle, UK). Recombinant NEP protein was a gift from Dr David Nanus (Cornell University, New York, USA). Primary cells (Lang *et al*, 1998) and P4E6, PNT1a, PNT2C2 cell lines (Maitland *et al*, 2001) were provided by York Cancer Unit. Matrigel, cell culture inserts and companion plates were obtained from Becton and Dickinson (Bedford, UK). Peptides and thiorphan were purchased from Sigma. The ECE-1 specific inhibitor, 4-chloro-*N*-[(4-cyano-3-methyl-1-phenyl-1*H*-pyrazol-5-yl)amino]carbonyl]benzene sulphonamide monosodium salt, referred to here as ECE-i, was described and characterised by Umekawa *et al* (2000). Endothelin-1 was obtained from The Peptide Institute (Barnet, UK).

### Cell Culture

Cell lines were routinely cultured in RPMI-1640 containing 2 mM L-glutamine and 10% FBS with the following exceptions: P4E6 cells were cultured in keratinocyte-SFM supplemented with bovine pituitary extract (25 *μ*g ml^−1^), recombinant epidermal growth factor (rEGF: 0.2 ng ml^−1^), 2 mM L-glutamine and 2% FBS. The mouse embryonic fibroblast cell line STO (Lang *et al*, 2001) was maintained in DMEM containing 2 mM L-glutamine and 10% (v v^−1^) FBS. All cells were routinely grown in antibiotic-free media at 37°C and 5% CO_2_. Primary stromal cells were maintained in RPMI-1640 supplemented with 2 mM L-glutamine, 10% (v v^−1^) FBS, 1% (v v^−1^) penicillin and 50 Units ml^−1^ streptomycin. Primary epithelial cells were maintained in keratinocyte serum-free medium supplemented with 2 mM L-glutamine, bovine pituitary extract (50 *μ*g ml^−1^), rEGF (0.2 ng ml^−1^) and 1% (v v^−1^) penicillin (50 Units ml^−1^) and streptomycin (50 *μ*g ml^−1^). The above cell culture reagents were all purchased from GIBCO BRL (UK).

### Invasion assay

The invasion assay was performed essentially as described by Lang *et al* (2000). The following supplements were added, either alone or in combination, to both STO (stromal) cell (bottom well) and epithelial cell (top well) media: NEP (50 *μ*g ml^−1^), thiorphan (1 *μ*M), bradykinin (100 nM), ET-1 (10 nM) and the specific ECE inhibitor ECE-i (100 nM). The invasion assay was incubated overnight at 37°C. Subsequently, the inserts were removed from the wells, washed in PBS, fixed in 100% methanol for 10 min and stained with 0.1% (w v^−1^) Crystal Violet (Sigma). Cells that had invaded to the underside of the inserts were counted by light microscopy. Four fields of view from each insert were counted. Two-tailed student *T*-tests were used to ascertain the statistical significance with a threshold of *P*<0.05.

### Immunofluorescence

Cells were grown to 60% confluency on sterile coverslips, washed twice with PBS and fixed and permeabilised for 10 min in methanol/acetone 50 : 50 at room temperature. Nonspecific binding sites were blocked for 30 min in blocking buffer (TBS, 1% (v v^−1^) normal goat serum and 0.2% (w v^−1^) gelatin). Primary antibodies were used at the following concentrations: ECE-1 (1 : 100), NEP (1 : 80). For negative controls, the primary antibody was replaced with pre-immune serum or IgG subclass antibody. Cells were washed three times in TBS and incubated for 30 min at room temperature with FITC conjugated anti-mouse IgG (1 : 1000) or FITC conjugated anti-rabbit IgG (1 : 1000) (Jackson ImmunoResearch Laboratory). Cells were washed three times in TBS, coverslips were inverted and mounted onto glass slides. Cells were examined using an Olympus IX70 inverted wide-field fluorescence microscope. Images were captured using Delta Vision from Applied Precision.

### Immunoblotting

Protein was isolated from membrane fractions from disrupted cells using N_2_ gas in a Parr cell disruption bomb (750 psi), resolved by SDS–PAGE, transferred to a nitrocellulose membrane (Invitrogen) and blocked in 0.1% Tween-20 in 10 mM Tris–HCl, pH 7.4 (TBST) with 5% (w v^−1^) milk powder. Membrane proteins were incubated with the following antibodies: ECE-1 (1 : 500) and NEP (1 : 100). Specific protein bands were detected using ECL reagents (Amersham Pharmacia).

## RESULTS

### NEP and ECE-1 protein expression in PC cell lines

**Figure 1:**
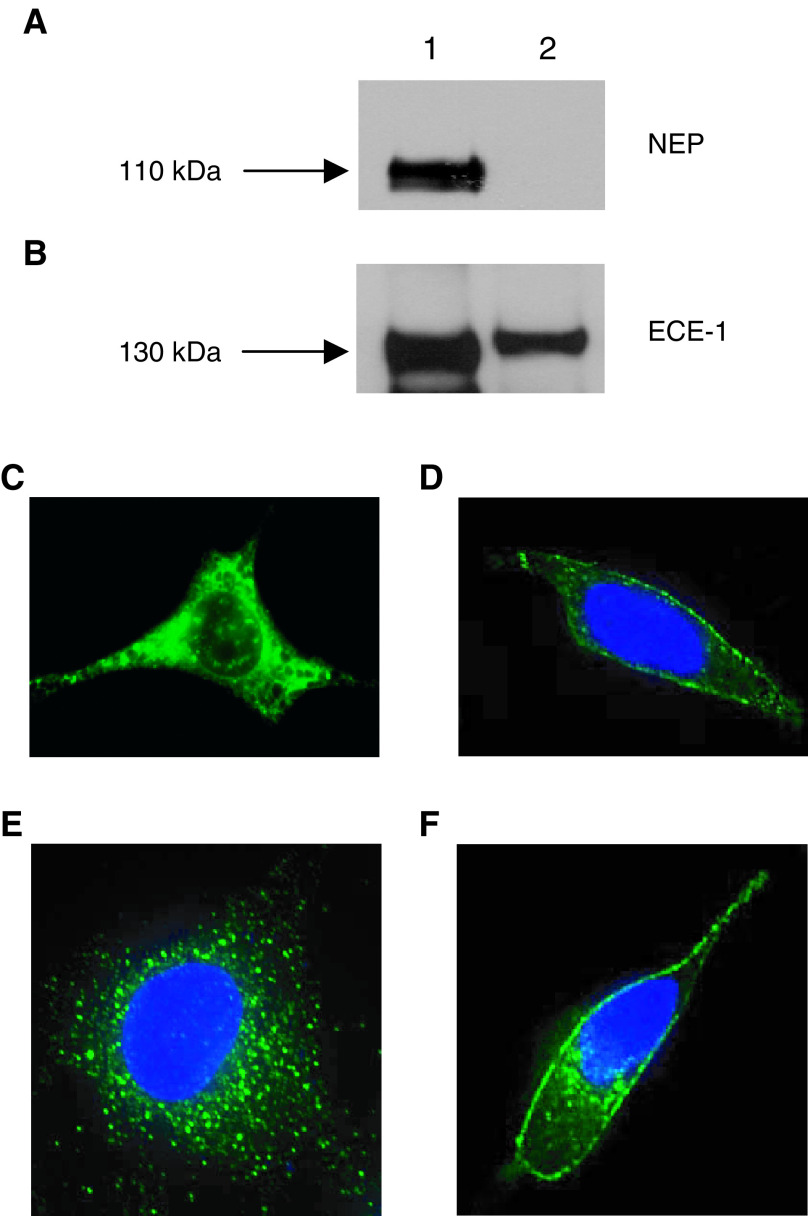
(**A**) Neutral endopeptidase and (**B**) ECE-1 protein expression in STO cells. Each lane contains 20 *μ*g of membrane protein. Lane 1: recombinant NEP (10 ng) or ECE-1c protein (500 ng), positive control. Lane 2 represents STO cells. Immunofluorescent staining determining ECE-1 protein expression and subcellular localisation in (**C**) STO cells, androgen-independent (**D**) PC-3 and (**E**) Du145 cells; (**F**) NEP protein expression and subcellular localisation in androgen-sensitive LNCaP cells.

Previous data (Usmani *et al*, 2002) have shown that high levels of NEP protein are expressed in LNCaP cells, whereas metastatic PC-3 and Du145 display low levels. In contrast, LNCaP cells have low ECE-1 protein expression while PC-3 and Du145 show comparatively high levels. A contrast in NEP and ECE expression is also evident in the transformed cell lines where PNT-1a cells show staining for NEP, whereas PNT2-C2 and P4E6 express ECE-1 protein. STO cells express comparatively high levels of ECE-1 (Figure 1B

) but do not express NEP (Figure 1A).

### Subcellular localisation of NEP and ECE-1 protein

Immunofluorescence analysis was used to investigate the subcellular distribution of NEP and ECE-1 protein in PC-3, Du145 and STO cells. Immunostaining of methanol/acetone-fixed STO cells revealed no staining with NEP (not shown), but bright diffuse staining with ECE-1 (Figure 1C). In PC-3 cells, ECE-1 protein staining was seen as a bright surface membrane and cytoplasmic fluorescence (Figure 1D), whereas Du145 revealed punctate staining throughout the cytoplasm (Figure 1E). Neither of the metastatic cells stained positive for NEP. In contrast, LNCaP cells revealed no staining with ECE-1 (not shown), but NEP protein staining was revealed predominantly at the cell surface (Figure 1F).

### PC cell invasion through Matrigel

**Figure 2:**
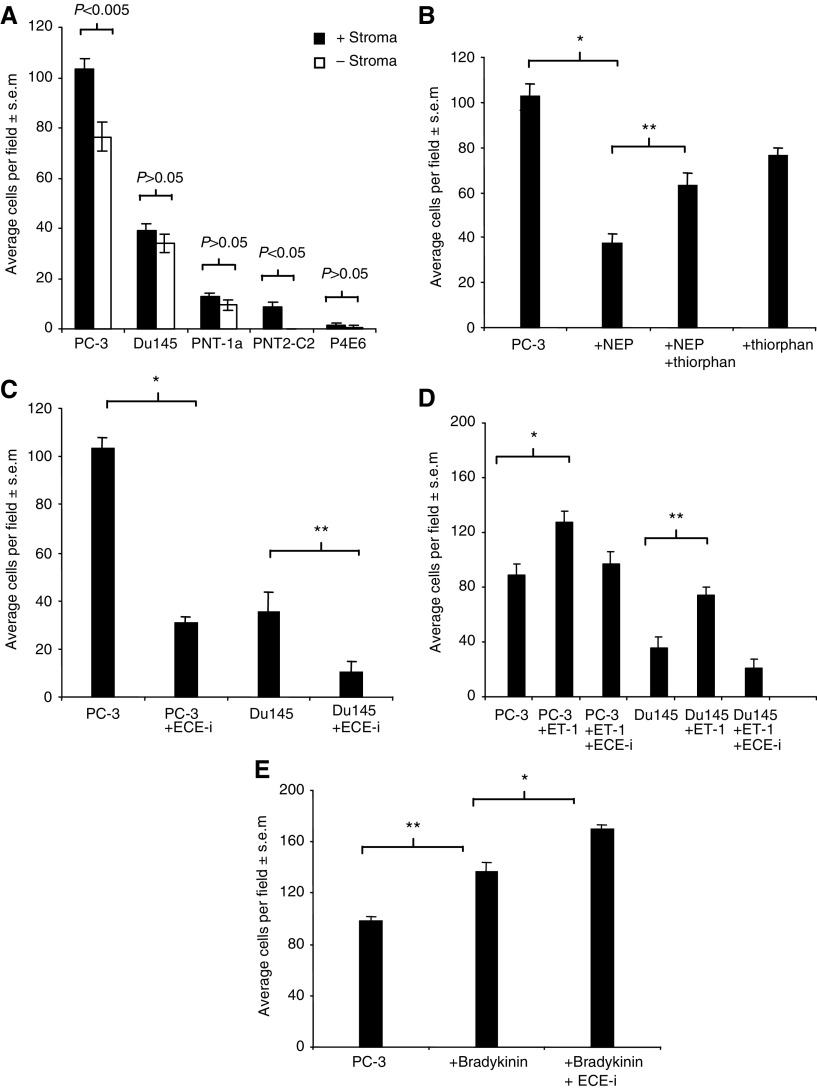
(**A**) Influence of stromal (STO) cells on invasion of established PC cell lines, PC-3, Du145, and virally transformed prostatic epithelial cell lines, PNT2-C2, PNT-1a and P4E6 through Matrigel. Error bars represent±s.e.m. of eight fields counted. (**B**) Invasion of PC-3 cells through Matrigel invasion chambers in response to the addition of recombinant NEP and thiorphan, a specific NEP inhibitor in the presence of STO. Neutral endopeptidase (50 *μ*g ml^−1^) and thiorphan (1 *μ*M) were added to both wells of the invasion chamber containing stromal cells and to the PC-3 cells (8 × 10^5^) on top of the Matrigel-coated insert. Each assay was incubated at 37°C for 24 h. Error bars represent±s.e.m. of eight fields. Data shown are representative of three separate experiments. ^*^P<0.001, ^**^P<0.005. (**C**) Invasion of PC-3 and Du145 cells through Matrigel in response to the addition of ECE-specific inhibitor (ECE-i) (100 nM), in the presence of STO. Treatments were added both to the STO on the base of the invasion chamber and to cells (8 × 10^5^) in the Matrigel-coated insert. The assay was incubated for 24 h. Bars represents the mean value of eight fields counted. Data shown are representative of three separate experiments. ^*^P<0.001, ^**^P<0.01. (**D**) Invasion of PC-3 and Du145 cells through Matrigel, following supplementation with ET-1 (10 nM) and ECE-i (100 nM) in the presence of stromal cells. Treatments were added to media above and below Matrigel. Each bar represents the mean value of eight fields counted. Data shown are representative of three separate experiments. ^*^P<0.001, ^**^P<0.01. (**E**) Invasion of PC-3 cells through Matrigel in response to the addition of bradykinin (100 nM) and the ECE-1 specific inhibitor ECE-i (100 nM) in the presence of STO. Treatments were added to the media above and below Matrigel. PC-3 cells were incubated in defined media for 24 h prior to the invasion assay. The assay was incubated for 24 h. Each bar represents the mean value of eight fields counted. Data shown are representative of three separate experiments. ^**^P<0.001, ^*^P<0.005.

The influence of stromal cells (STO) on the ability of the PC cell lines to invade Matrigel was measured using indirect co-culture. The data show that PC invasion is increased in the presence of stromal cells. PC-3 and Du145 were the most invasive in comparison to the transformed prostate epithelial cells lines, PNT-1a, PNT2-C2 and P4E6, which showed much less invasion both in the presence or the absence of stromal cells. Overall, a general trend of increased invasion was observed in the presence of stromal cells, with significant increases for PC-3 and PNT2-C2 (Figure 2A

).

### Modulation of PC cell line invasion by mitogenic peptide inactivation

The addition of recombinant NEP resulted in a decrease of approximately 50% in PC-3 cell invasion in the presence of STO cells (Figure 2B). The inclusion of thiorphan (1 *μ*M), a specific NEP inhibitor, partially reversed this effect. Thiorphan alone had no significant effect on invasion when compared with NEP plus thiorphan (Figure 2B). In the absence of stromal cells, there was no significant difference in PC-3 invasion between control and treated wells (data not shown). Similar results were obtained using Du145 cells (data not shown). Inhibition of ECE-1 activity reduced PC-3 invasion by approximately 70% in the presence of stromal cells (Figure 2C). In the absence of STO, there was no significant change in the invasive capacity of treated and untreated PC-3 cells (data not shown). Similar results were obtained for Du145 with ECE-1 inhibition, resulting in a 50% decrease in invasion (Figure 2C).

### Effect of mitogenic peptides on cell invasion through Matrigel

The addition of ET-1 resulted in an increase of approximately 50% in PC-3 cell invasion through Matrigel in the presence of stromal cells (Figure 2D). The inclusion of an ECE inhibitor with ET-1 reduced invasion (Figure 2D) by inhibiting endogenous stromal ECE activity, since no significant effect was observed in the absence of stromal cells (data not shown). Supplementation with ET-1 resulted in 100% increase in Du145 invasion through Matrigel in the presence of stromal cells (Figure 2D). The simultaneous addition of ET-1 and an ECE inhibitor reduced invasion to below control levels, but not as much as ECE inhibitor alone (Figure 2C). Supplementation of serum-free media with the mitogenic peptide bradykinin increased PC-3 invasion by approximately 40% in response to indirect co-culture (Figure 2E). The concomitant addition of bradykinin and ECE-1 inhibitor resulted in an approximately 70% increase in PC-3 invasion, consistent with a reduced ability of ECE-1 to inactivate bradykinin. No significant effect was observed in the absence of stromal cells (data not shown).

### Effects of primary stromal cultures on PC-3 invasion

**Figure 3:**
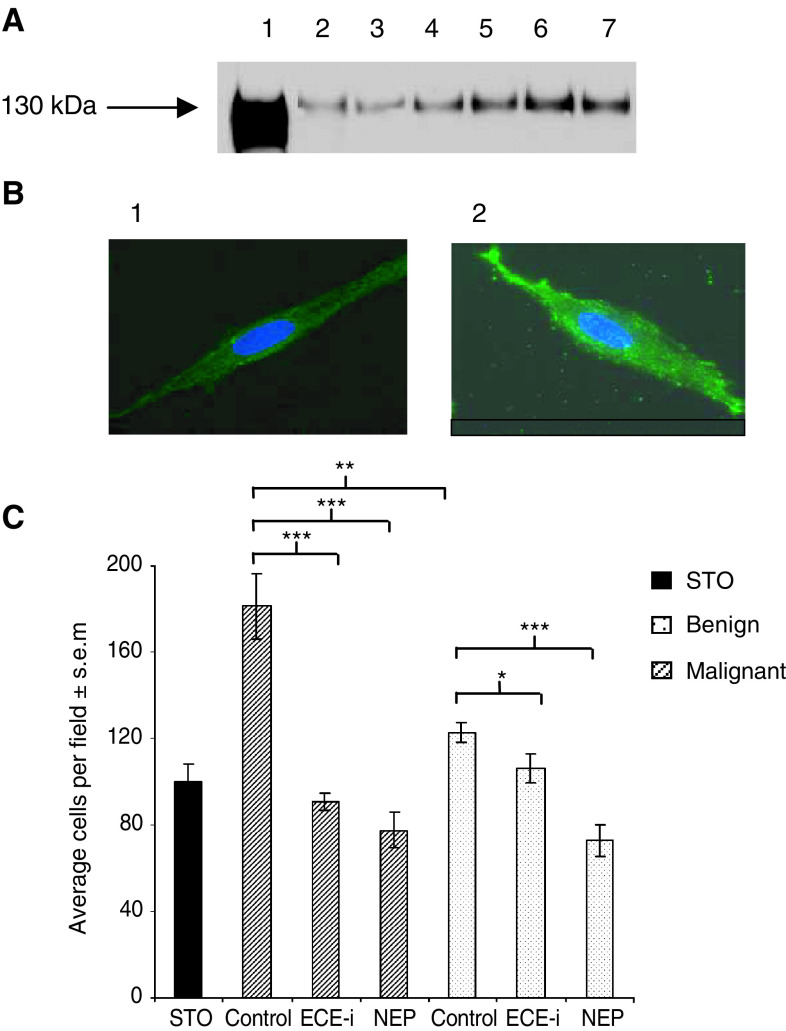
(**A**) ECE-1 protein expression in primary stromal cells derived from malignant and benign tissue. Each lane contains 20 *μ*g of membrane protein. Lane 1: ECE-1c protein (500 ng), positive control. Lanes 2, 3 and 4 represent primary stromal cells derived from benign tissue. Lanes 5, 6 and 7 show primary stromal cells derived from malignant tissue. (**B**) Immunofluorescent staining determining ECE-1 protein expression and subcellular localisation in (1) benign and (2) malignant primary stromal cells. (**C**) Invasion of PC-3 cells through Matrigel in response to the addition of recombinant NEP and ECE-i in the presence of primary stromal cells derived from malignant and benign tissue. Error bars represent±s.e.m. of eight fields. ^*^P<0.05, ^**^P<0.005, ^***^P<0.001.

Endothelin-converting enzyme-1 protein expression was determined in primary stromal cells derived from malignant and benign tissue by Western blotting. Higher levels of ECE-1 protein are expressed in malignant primary stromal cells than in benign primary stromal cells (Figure 3A

). Endothelin-converting enzyme-1 staining of malignant primary stromal cells was seen as bright surface membrane and punctate cytoplasmic fluorescence (Figure 3B(2)). Benign and malignant primary stromal cell staining differ in intensity only (Figure 3B(1)). The effects of either ECE-1 inhibition or recombinant NEP activity on PC-3 invasion in the presence of malignant and benign primary stromal cells were examined. Results indicate that stromal cultures from malignant and benign tissue can stimulate the invasion of PC-3 cells by approximately 80 and 20%, respectively, beyond the influence of STO cells (Figure 3C). Endothelin-converting enzyme-1 inhibition reduced the influence of malignant stroma on PC-3 invasion by approximately 50%, but had a less significant effect in the presence of benign stromal cells, reflecting the lower levels of ECE-1 activity in benign cells. Interestingly, the addition of recombinant NEP resulted in an approximately 55% and an approximately 40% decrease in PC-3 invasion in the presence of malignant primary stroma and benign stroma respectively. This might be anticipated given that NEP can inactivate a range of peptide mitogens including ET-1.

## DISCUSSION

Many human cancers generate a reactive stroma that promotes tumour invasion, progression and metastasis. An investigation into the properties of reactive stroma can reveal considerable insights into the risk factors for tumorigenicity as reviewed recently (Tuxhorn *et al*, 2001). During prostate carcinogenesis, the stroma undergoes progressive alterations to develop a phenotype that co-operates with the tumorigenic epithelial cells to help promote cell motility and invasion (Tuxhorn *et al*, 2001). Such alterations include matrix remodelling enzymes (Egeblad and Werb, 2002). Our study reveals that alterations in the balance of two membrane-bound metallopeptidases, NEP and ECE-1, can produce stroma that modulates the invasive potential of prostate cancer epithelial cells. Endothelin-converting enzyme-1 is expressed in the stromal compartment, where it generates an active endothelin (ET-1) peptide and metabolises other peptides including bradykinin. A mitogenic role for small regulatory peptides such as ET has been implicated in PC progression, with elevated levels found in patients whose tumours have become androgen-independent (Nelson *et al*, 1995). Neutral endopeptidase is expressed in the epithelial layer and functions to cleave and inactivate a number of small mitogenic peptides, including ET-1 and bradykinin. In metastatic PC, NEP is dramatically downregulated and its loss consequently contributes to tumour progression (Papandreou *et al*, 1998). Here, we have utilised indirect stromal–epithelial co-culture techniques to demonstrate the influence of a stromal cell line (STO) and primary stromal cultures on the ability of metastatic PC cell lines to invade Matrigel as a consequence of relative NEP and ECE-1 activity. Our data show that malignant PC-3 and Du145 cells have a greater natural propensity to invade Matrigel than the transformed but nonmalignant prostate epithelial cell lines PNT2-C2, PNT-1a and P4E6. This greater propensity is likely due to a host of genetic alterations, which favour invasion and metastasis. This invasive phenotype can be further augmented in the presence of stromal cells as a consequence of their high ECE-1 expression and ET-1 availability to the tumour. The role of stromal involvement during PC progression has been reported elsewhere (Lang *et al*, 2000), and this is consistent with recent observations that epithelial–stromal interactions are critical determinants of tumour cell behaviour. Ramaswamy *et al* (2003) compared the geneexpression profiles of metastatic adenocarcinomas with their primary counterparts and found that a considerable proportion of the refined gene-expression signature associated with metastasis was derived from the nonepithelial components of the tumour.

In the rat prostate gland, big-ET, the inactive precursor of ET, was observed in the stroma and co-localised with *α*-actin of smooth muscle fibroblasts (Ventura and Salamoussa, 2002). Endothelin-converting enzyme-1 has also been shown to co-localise with *α*-actin in human vascular smooth muscle cells (Barnes and Turner, 1999). This implies that ET-1 is synthesised on demand from bigET in the stroma of the prostate. We have found bigET-1 in primary stromal cells (data not shown). Exogenous ET-1 can further increase invasion of PC-3 and DU145 in the presence of stromal cells, and this effect can be partially reversed by inhibiting endogenous stromal ECE activity, demonstrating the very high demand of PC cells for mitogenic peptides. Similar results were observed with the mitogenic peptide bradykinin, an ECE substrate, with the exception that addition of an ECE-1 inhibitor further increased invasion by inhibiting ECE hydrolysis of bradykinin.

The most dramatic effect was seen with primary stromal cells of benign and malignant origin. Our current data on a small number of samples revealed that indirect co-culture with stromal cultures from both benign and malignant tissue stimulated the invasion of PC-3 cells beyond levels previously observed for STO cells. Furthermore, malignant stroma recruited more invasive epithelial cells than benign stroma, indicating that the neoplastic process alters stromal cell activity in such a way as to maximally enhance invasion. Similar findings have been reported in the motility of epithelial response to indirect co-culture (Lang *et al*, 2000).

Immunocytochemical analysis has revealed differences in ECE-1 localisation between PC-3 and DU145 cells and these patterns may relate to specific isoforms of ECE-1. Any significance of specific isoforms of ECE in the invasive process is currently being explored. In contrast to the ECE-1 data, we observed that invasion of the androgen-independent cell lines PC-3, Du145, could be decreased by the addition of recombinant NEP and that these effects could be neutralised by the addition of thiorphan, an NEP-specific inhibitor. This correlation in PC cells of loss of NEP expression and cell migration is explained by the catalytic capability of NEP to inactivate mitogenic peptides such as ET-1 and bradykinin which promote cell migration. This is consistent with the findings of Suzuki *et al* (2001) in human endometrial cancer.

Recent data reveal that NEP has signalling properties independent of its enzyme activity (Sumitomo *et al*, 2000). Could these signalling properties extend to coordinate ECE expression? Or does ECE have unique signalling properties of its own? Answers to these questions are currently being addressed and may provide further insight into the mechanisms behind the observations reported in this study.

The current study concludes that downregulation of NEP in prostate cancer epithelial cells allows mitogenic peptides to impart growth signals to the developing tumour, while in the surrounding prostate stroma elevated ECE-1 provides a local source of active ET-1 which encourages invasion. It may be possible therefore to inhibit ECE activity in the stroma and suppress the overall growth of the tumour. Concomitant delivery of recombinant NEP would neutralise the effects of bradykinin and other peptides and might result in significant tumour regression. A better understanding of the relative roles of NEP and ECE in the development, invasion and progression of prostate tumours will help in the potential design of useful therapeutic agents to treat prostate cancer.
